# The behavioral and social drivers of HPV vaccination among parents and young people in Indonesia: a scoping review

**DOI:** 10.1007/s10552-025-02027-x

**Published:** 2025-07-02

**Authors:** Aisya Athifa, Yasmin Mohamed, Isabella Overmars, Margie Danchin, Jessica Kaufman

**Affiliations:** 1 School of Population and Global Health, The University of Melbourne, Parkville, VIC Australia; 2 Vaccine Uptake Group, Murdoch Children’s Research Institute, Parkville, VIC Australia; 3 Department of Paediatrics, The University of Melbourne, Parkville, VIC Australia

**Keywords:** Cervical cancer, HPV vaccination, Social and behavioral drivers, Indonesia, Scoping review

## Abstract

**Purpose:**

The Indonesian Government launched the national human papillomavirus (HPV) vaccination program in August 2023, reaching 90% coverage for both doses. This scoping review explored the behavioral and social drivers of HPV vaccination among parents and young people in Indonesia.

**Methods:**

We searched four databases for primary quantitative, qualitative, and mixed-method studies in English or Bahasa Indonesia assessing behavioral and social drivers of HPV vaccination in Indonesia. Participants were parents and young people under 24. The quality was appraised with the Mixed Methods Appraisal Tool. Narrative synthesis was conducted to summarize findings according to the World Health Organization’s Behavioral and Social Drivers (BeSD) of vaccination framework.

**Results:**

Eighteen studies were included. Drivers were mapped across the BeSD domains: thinking and feeling, social process, motivation, and practical issues. The majority were related to what people think and feel including low knowledge and awareness of HPV disease and vaccines despite high motivation to vaccinate. This review identifies the importance of HPV vaccines’ halal-haram status. Spouses and teachers were the most cited influencers in vaccine decision-making not healthcare providers. Puskesmas was the preferred vaccination location and concerns about vaccine costs were frequently mentioned.

**Conclusion:**

This review identifies the main drivers of HPV vaccination among parents and young people in Indonesia to optimize HPV vaccine uptake as the national rollout is expanded. Clear communication about the halal-haram status of HPV vaccines, involvement of parents, family, teachers, and trusted community members to communicate about HPV vaccines and ensuring HPV vaccine accessibility outside schools are needed.

**Supplementary Information:**

The online version contains supplementary material available at 10.1007/s10552-025-02027-x.

## Introduction

Cervical cancer burden is high in Indonesia, being the second most common cancer among women, with 36,633 cases identified each year [1]. Greater than 70% of cases identified are in advanced stages, resulting in 21,003 deaths annually [1, 2]. Human papillomavirus (HPV) infection is a primary cause of cervical cancer [3, 4], with HPV types 16 and 18 responsible for 70% of cervical cancer cases worldwide [5–7]. The prevalence of HPV infection is high among young women, peaking between 18 and 30 years old [3], while the diagnosis of cervical cancer occurs later in a woman’s life [3]. The HPV vaccine can prevent most cases of cervical cancer if given in early adolescence [3, 6, 8].

The Indonesian Government’s commitment to reduce the country’s cervical cancer burden is demonstrated by the inclusion of HPV vaccines onto the national immunization program (NIP), as recommended by the World Health Organization's (WHO) Global Strategy to Accelerate the Elimination of Cervical Cancer [9–11]. HPV vaccine introduction began in 2016 as a pilot program in Jakarta [2] and was expanded to 20 districts and cities by 2021 [2, 12]. The Indonesian Government officially launched the nationwide expansion in August 2023 by delivering HPV vaccines free of charge for girls in 5th and 6th grade of elementary school (aged 11 and 12 years) [2]. The quadrivalent vaccine is provided in two doses with a 12 month interval [13, 14]. School-based delivery has been implemented through the School Immunization Month or Bulan Imunisasi Anak Sekolah [2, 12, 15]. Bulan Imunisasi Anak Sekolah is conducted each year in August and November with the vaccine delivered by healthcare providers from the puskesmas (public health centers) [16]. The pilot program in Indonesia in 2017 and 2018 reached > 90% of targeted girls in the piloted districts for both doses [2, 16] but the national HPV vaccine coverage in 2022 was only 29% first dose and 7% second dose [17], well below WHO's target of 90% for adolescent girls [9]. No data have yet been reported on national vaccine coverage after the launch of the nationwide program.

There are many reasons for lower HPV vaccine uptake compared to routine childhood vaccines. Parental consent is needed for HPV vaccine administration in most settings and requires the return of signed consent forms to school [18–20], including in Indonesia [21]. This can create complexity or a disconnect between parents and young people in the HPV vaccination implementation: adolescents are old enough to have an opinion about vaccination, but their parents dictate the vaccine decision, and are not present during vaccine administration [18]. The fact that HPV vaccination protects against a sexually transmitted infection (STI) is a sensitive topic in many countries with specific cultural implications and often creates stigma associated with promiscuity [22, 23]. Furthermore, cervical cancer is a slowly progressive disease, with the preventative benefit of vaccination experienced many years after vaccination [3, 24]. These factors make the decision-making process for caregivers and young people around HPV vaccination more complex than for other routine childhood vaccines and underpin the need to understand the social and behavioral drivers of HPV vaccination.

**Fig. 1:**
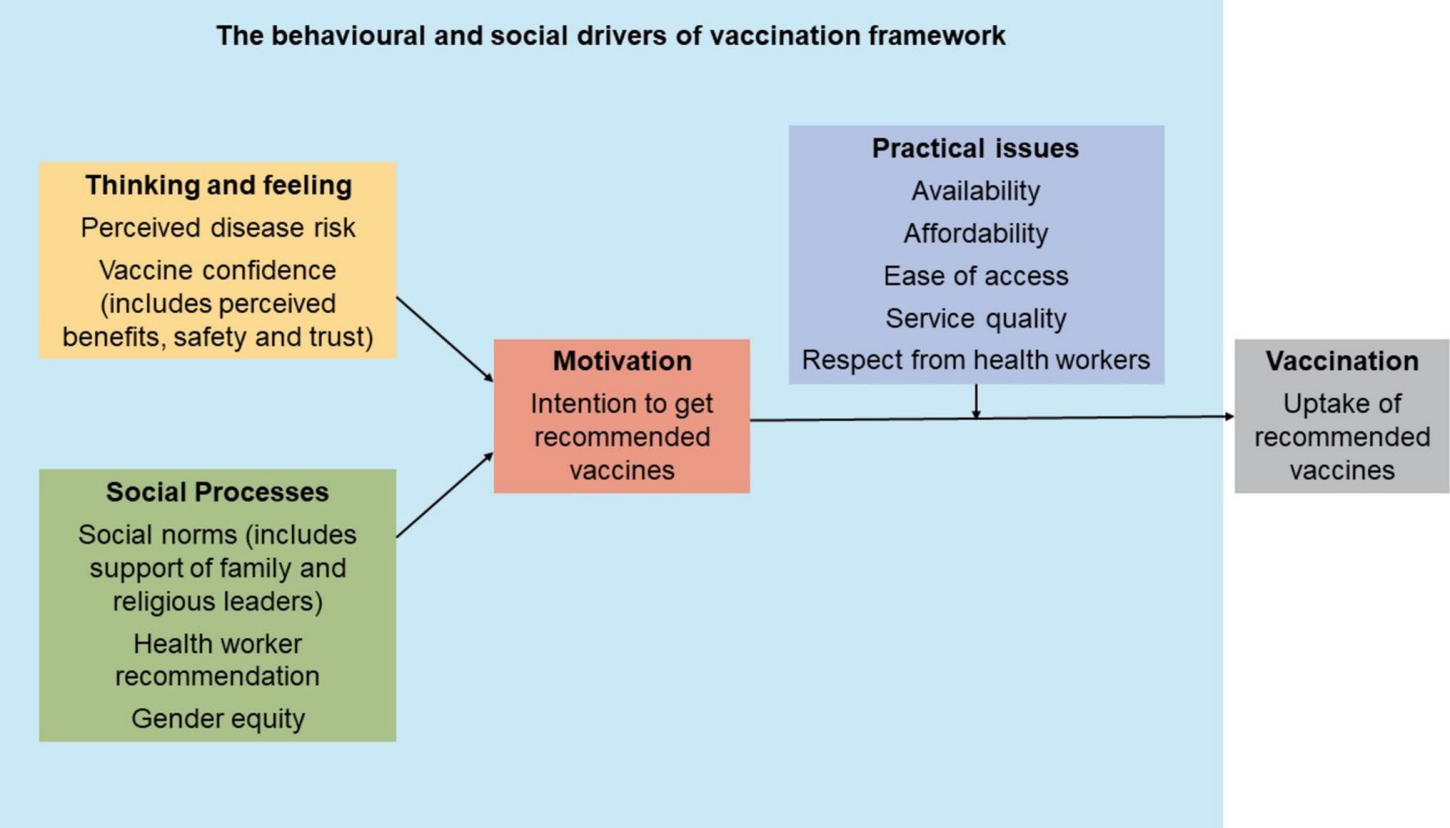
The BeSD framework [25]

The drivers associated with HPV vaccination can be mapped to the domains of the WHO Behavioral and Social Drivers (BeSD) of vaccination framework including thinking and feeling, social process, motivation, and practical issues (Fig. 1) [25]. The BeSD framework outlines modifiable access and acceptance barriers to improve vaccine uptake [25]. With the high burden of cervical cancer in Indonesia, HPV vaccination is critical to the country’s cervical cancer prevention efforts. The national expansion of the HPV vaccination rollout, along with Indonesia’s unique cultural context, highlight the importance of understanding and addressing the drivers of HPV vaccination in the country. This is the first review to explore the behavioral and social drivers of HPV vaccination among parents and young people in Indonesia to inform HPV vaccination policy and practice and increase vaccine uptake.

## Methods

This scoping review was conducted with guidance from the Joanna Briggs Institute (JBI) Manual for Evidence Synthesis [26]. It is reported according to The Preferred Reporting Items for Systematic Review and Meta-Analyses extension for Scoping Reviews (PRISMA-ScR) checklist [27] and was registered in the International Prospective Register of Systematic Reviews (PROSPERO) (CRD42024525525). The PRISMA-ScR checklist and PROSPERO registration is attached to Supplementary Files 1 and 2, respectively.

### Eligibility criteria

Studies were included if they were primary quantitative, qualitative, or mixed-method studies that assessed behavioral and social drivers of HPV vaccination in Indonesia. Participants included in the review were parents and young people of any age up to 24 years old [28] to align with the Lancet Child and Adolescent Health’s expanded definition of adolescence which is more representative of current adolescent growth and development [28]. This age group also represents the primary and secondary target population of HPV vaccination based on WHO recommendations [29]. The review included published studies conducted in Indonesia. Where studies were conducted in more than one country, data in Indonesia was separated and included in the review. Studies published in the English language and Bahasa Indonesia with no limitation of publication dates were included.

### Search strategies

The databases searched were MEDLINE (Ovid), EMBASE, PubMed, and Global Health. The search strategies were developed with assistance of a librarian. The search strategies, number of articles found, and search date for each database are presented in Supplementary File 3.

### Selection process

The search results from the databases were exported to EndNote [30], with duplicates removed. Title and abstract screening was conducted independently by two reviewers (AA, YM, and/or IO) in Covidence [31]. Full articles were retrieved and screened independently by two reviewers (AA, YM, and/or IO) based on the inclusion criteria, documenting reasons for exclusion. Any disagreement was resolved through a discussion with the fourth author (JK).

### Data extraction

The data extraction template was developed using an Excel spreadsheet and discussed among reviewers to reach agreement on which content was to be extracted from included studies. It was piloted and adapted during the data extraction process. Extracted data included study authors, year of publication, study design, study aim, participant characteristics, sample size, measurement scale used, data on frequency including numbers and percentages, association with intention or uptake using odds ratio, and description of themes with relevant quotes for the qualitative studies. The main results were categorized based on the WHO BeSD domains. The data extraction template is presented in Supplementary File 4.

### Critical appraisal of evidence

Included studies underwent a critical appraisal using the mixed-method assessment tool (MMAT) [32] by one reviewer (AA) and checked by another reviewer (YM, IO). Disagreements were discussed with the fourth reviewer (JK). We did not exclude studies with low methodological quality or assess study risk of bias.

### Data analysis

A narrative synthesis was conducted to present the review findings [33, 34]. Data were categorized based on study design, population, and results, and presented in a descriptive format. We identified themes within each BeSD domain and then synthesized the findings and studies contributing data to each domain. Where identified, we also reported potential trends in data within each domain based on study publication date. The studies were analyzed by one reviewer (AA); the analysis was reviewed by the senior author (JK).

## Results

### Study selection

**Fig. 2:**
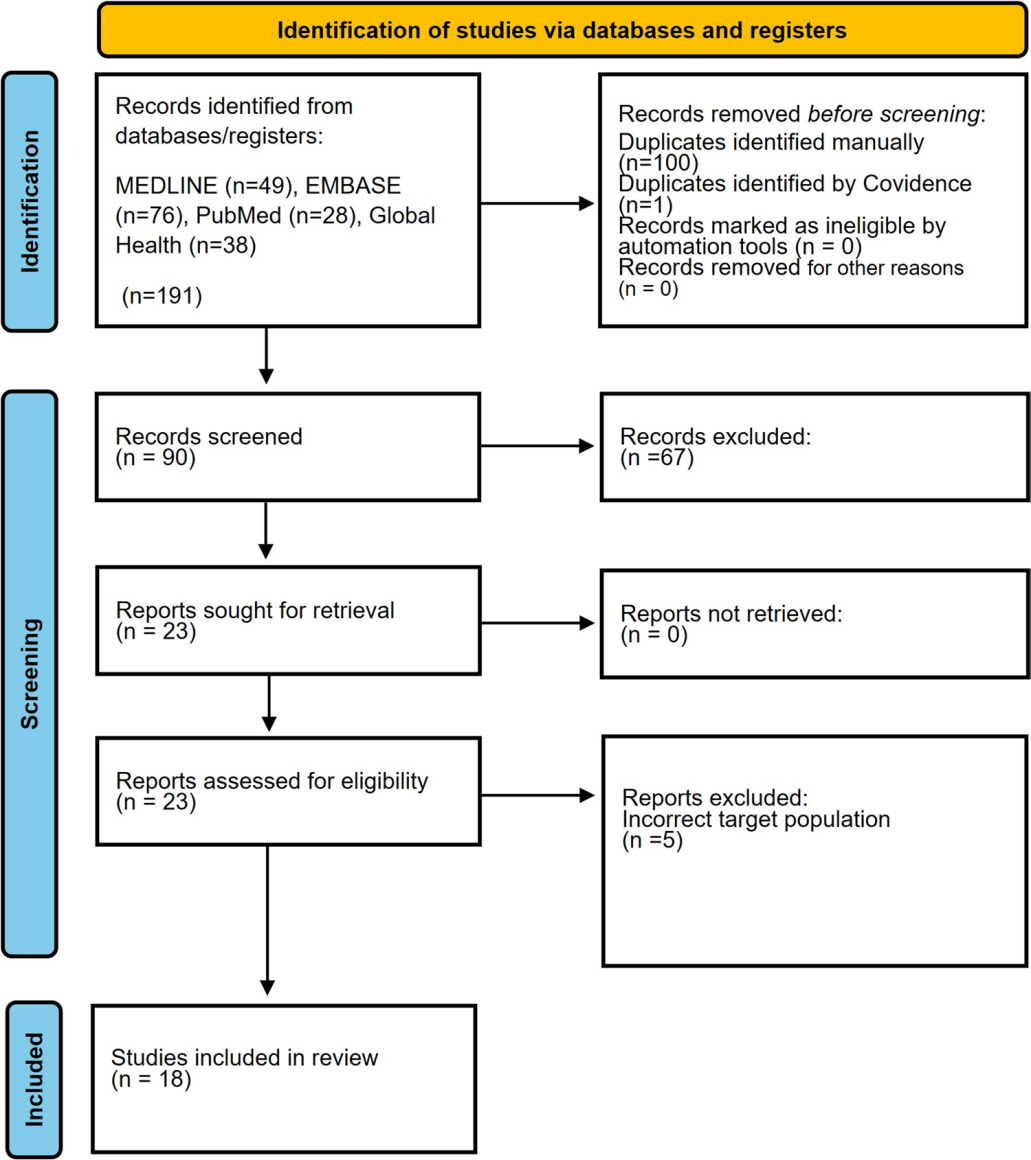
PRISMA-ScR diagram

The PRISMA-ScR diagram is shown in Fig. 2. The initial searched retrieved 191 studies across four databases, with 23 studies reviewed in full text and 18 studies included in the final review (see excluded studies with reasons in Supplementary File 5).

### Study characteristics

Table 1 summarizes the characteristics of the included studies which focused on HPV vaccination only and were published in English. Fourteen studies were quantitative [21, 35–47], three studies were qualitative [48–50], and there was one mixed-method study [51]. The studies were published between 2011 and 2023, with all conducted prior to the national HPV vaccination rollout and all but two conducted before the COVID-19 pandemic. Among the 14 quantitative studies, 12 were cross-sectional surveys [21, 35–42, 45–47] and two were interventional studies [43, 44]. Only baseline characteristic data were extracted from these two intervention studies.

**Table 1:** Summary of included studies

Author (year published)	Provinces	Study design	Data collection method	Year of data collection	Participants	Publication type	BeSD Domains reported*			
							T&F	SP	M	PI
Endarti (2018) [42]	Yogyakarta	Quantitative, CS*	Self-administered survey	2014	Undergraduate female students from health and non-health faculty, female parents	Full text	X		X	X
Frianto (2022) [41]	West Java	Quantitative, CS	Self-administered survey	Not mentioned	Parents	Full text	X	X	X	X
Jaspers (2011) [35]	South Kalimantan, North Sulawesi,Bali, North Sumatra, and East Java	Quantitative, CS	Interviewer-administered survey	2009	Parents	Full text	X	X	X	X
Kristina (2020) [36]	Yogyakarta	Quantitative, CS	Interviewer-administered survey	2019	Parents	Full text	X	X	X	X
Lismidiati (2020) [50]	Yogyakarta	Qualitative	Focus group discussion	Not mentioned	Junior high school female students and parents	Full text	X	X		X
Lismidiati (2021) [44]	Yogyakarta	Quantitative, quasi-experimental with non-equivalent (pre-test and post-test) control group design	Self-administered survey	2018–2019	Junior high school female students	Full text	X			
Lismidiati (2022) [40]	Yogyakarta	Quantitative, CS	Self-administered survey	2018	Junior high school female students	Full text	X	X		
Prayudi (2015a) [46]	Bali	Quantitative, CS	Self-administered survey	Not mentioned	Parents	Abstract	X			X
Prayudi (2015b) [47]	Bali	Quantitative, CS	Self-administered survey	Not mentioned	Adolescent girls	Abstract	X			
Prayudi (2016) [37]	Bali	Quantitative, CS	Self-administered survey	2016	Junior high school female students	Full text	X			
Sitaresmi (2020) [43]	Yogyakarta	Quantitative, pre- and post-test study	Self-administered survey	2017	Parents	Full text	X			
Spagnoletti (2019) [49]	Central Java	Qualitative	Focus group discussion and semi-structured interview	2015	Married men and women with children	Full text	X	X	X	X
Susanto (2020) [51]	Bali	Mixed method	Self-administered survey and in-depth interview	2018	Parents, 5th grade schoolgirls	Full text	X	X	X	X
Wibisono (2017) [39]	Banten	Quantitative, CS	Interviewer-administered survey	Not mentioned	Undergraduate medical and nursing students (female and male)	Abstract	X			
Widjaja (2017) [38]	Not mentioned	Quantitative, CS	Interviewer-administered survey	Not mentioned	Undergraduate medical students (female and male)	Abstract	X			
Wijayanti (2021) [21]	Jakarta	Quantitative, CS	Self-administered survey	2019	Parents	Full text	X	X	X	X
Wijayanti (2023) [48]	Jakarta	Qualitative	Semi-structured interview	2019	Parents	Full text	X	X	X	X
Winarto (2022) [45]	Jakarta	Quantitative, CS	Self-administered survey	2020–2021	Young people (female and male)	Full text	X			

*CS: Cross−sectional, T&F: thinking and feeling, SP: social process, M=Motivation, PI=Practical Issue

Eight studies focused on parents [21, 35, 36, 41, 43, 46, 48, 49], seven studies focused on young people [37–40, 44, 45, 47], and three studies focused both on parents and young people [42, 50, 51]. The young people in the included studies were aged 9 to 24 years old, with most in the adolescent age range (age 12 to 16, with mean age 13) in junior high school. Three studies included undergraduate students studying medicine (median 19) [38], medicine or nursing (median age 19) [39], or other subjects (median age range 16–20) [42]. One study focused on schoolgirls in primary school (mean age 10) as a primary target population of the national HPV vaccination program [51]. All studies were conducted in Indonesia, none were conducted in multiple countries. The location of the studies was across several provinces in Indonesia, with most of the studies conducted in provinces within Java Island.

### Quality assessment of included studies

Most studies only met two out five (40%) of the criteria, while only four studies met all five criteria [21, 37, 43, 48] (see Table 2).

**Table 2:**
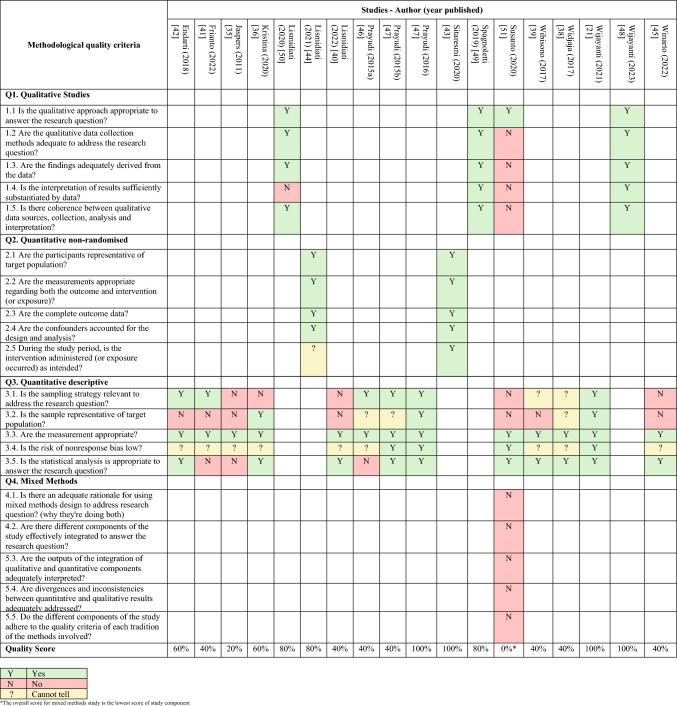
MMAT assessment

## Summary of findings

Studies were summarized within four WHO BeSD domains, with sub-categories identified within each domain (Table 3). Thinking and feeling was the most commonly assessed category of drivers.

**Table 3:** Identified sub-categories for each domain and number of studies

Domain	Sub-categories	Number of studies
Thinking and feeling	Awareness	10
	Knowledge	15
	Perception of HPV vaccination information	5
	Vaccine confidence	9
	Concerns about vaccines	9
	Disease perspective	7
	Attitudes	6
Social process	Influence of religion	7
	People who could influence vaccine decision	8
Motivation domain	Acceptance/willingness/intention	7
Practical issues	Affordability	10
	Accessibility	4

### Thinking and feeling domain

#### Awareness

Ten studies assessed awareness by asking parents [35, 36, 42, 43, 46, 49, 50] and young people [37, 42, 44, 47, 50] whether they had heard of cervical cancer, HPV infection, or HPV vaccination. Cervical cancer awareness was high in both groups [35–37, 42, 42, 43, 46, 47, 49, 50]. Awareness of HPV infection was lower [35–37, 42, 46, 47, 49, 50], and awareness of HPV vaccine was the lowest among both parents (range 15.8% to 44.1%) [35, 36, 42, 43, 46] and young people (range 24.1% to 68.2%) [37, 42, 44, 47]. Two qualitative studies also found that parents and young people had either never heard of HPV vaccination, or had only just heard of it [49, 50]. However, one quantitative study showed high awareness of HPV vaccine among both groups [46, 47], and one qualitative study showed that young people had heard of it [50]. There was no discernible pattern in the level of awareness of cervical cancer, HPV infection, and HPV vaccine among parents and young people over the period of time covered by the included studies.

#### Knowledge

Knowledge was the most frequently reported driver in all the studies, with eight studies focused on parents [35, 41–43, 46, 48–50], five studies focused on young people [37–40, 47], and two studies focused both [42, 51]. Seven of eight studies showed low knowledge among parents [35, 36, 43, 46, 48–50]. Only one study, conducted relatively recently in 2022, showed good knowledge, with almost 90% of parents able to answer most true or false questions correctly [41]. “Good knowledge” (undefined) was reported among young people in two quantitative studies [38, 40]. One study found medical students had better knowledge (undefined) than nursing students [39], and two showed that knowledge was better in adolescents compared to parents [42, 51]. One study indicated low knowledge among young people, with total mean score for cervical cancer and HPV vaccination knowledge below midpoint [47]. Higher knowledge was associated with vaccination status in two studies [37, 38] and with self-efficacy to obtain vaccination in third study [40]. However, three studies showed no association between higher knowledge and HPV vaccine acceptance [35, 41, 42].

#### Perception of HPV vaccination information

Five studies assessed parents’ perceptions about provided information related to HPV vaccination. Insufficient information was cited by parents as a reason why they were not or may not be willing to vaccinate their daughter [35, 36, 41]. One study showed that parents lacked information about vaccine types, vaccine benefits, and vaccine schedule [51], with another study reporting parents wanted more information before making their decision to vaccinate their daughter [48]. While parents consistently reported a lack of provided information, the percentage of parents citing this was lower in the more recent years of studies.

#### Vaccine confidence

Nine studies assessed vaccine confidence, defined as belief that HPV vaccine is effective, important, safe, and/or a good way to prevent cervical cancer. Most parents and young people believed that the HPV vaccine is effective, with more than 50% parents across five studies [35, 36, 41, 42, 46], and 84% students affirming this belief [42, 47]. Parents in four studies agreed that HPV vaccination is important [36, 49] or cited vaccine importance as a reason to vaccinate their daughter [35, 41]. More than 50% of parents in two studies believed that HPV vaccine is safe [41, 43]. Safety of HPV vaccine was also cited as a factor to enhance vaccine uptake among parents and students [42]. Almost 80% parents agreed or strongly agreed that HPV vaccine is useful to prevent cervical cancer [43] or cited this as a reason to vaccinate their daughter [35, 41]. Vaccine confidence among parents was lower in earlier studies, relatively high in studies from 2015 to 2019, and lower in the most recent study from 2022. Vaccine confidence among young people was only assessed in the earlier years of studies.

#### Vaccine concerns

Concern about the HPV vaccine was assessed in nine studies, focused on parents [35, 36, 41, 46, 48, 51], young people [47, 50], and both groups [42]. Fear of unspecified “vaccine side effects” was cited as a reason for rejecting or potentially rejecting HPV vaccination among parents in four quantitative studies (range 4 to 66.67%) [35, 36, 41, 42], a finding that was echoed in a qualitative study [48]. One study that assessed willingness to pay for HPV vaccination among parents found that 43.1% were unwilling to pay because of fear of side effect of the vaccine [46]. Fear of side effects of HPV vaccine among young people was low (3.1%) [42], but it was noted in a qualitative study, with one participant saying: “I’m worried about the vaccine’s expiry and side effect” [50]. Two studies assessing fear of potential harm from HPV vaccine among parents and students by using a mean score reported similar scores in both groups, but because the scale was not described it is not possible to determine whether the means were high or low [46, 47]. There were no temporal patterns in the rate of vaccine concerns among parents and young people.

#### Disease perspective

Seven studies assessed perspectives on disease severity among parents [35, 36, 41, 43, 49] and young people [45, 47]. Belief that cervical cancer is a serious disease was high among parents in three quantitative studies (range 87.8% to 99.1%) [35, 36, 43]. One study assessed this belief among young girls calculated with a mean score but there was insufficient information to determine whether the mean was high or low [47]. Belief that women are at risk developing cancer was high among parents, with 72.6% of parents in one study agreeing [43] and 99% of parents stating they were afraid or very afraid that their daughter would develop cervical cancer in the future [35]. One study showed that younger people were significantly more likely to fear their close family or partner getting cancer [45]. However, the perception that women are at risk of getting HPV infection was low, with only 40.3% of parents agreeing or strongly agreeing with this belief [43]. One study assessed that adolescent girls did not perceive themselves to be at risk of developing cervical cancer or HPV infection with a mean score 1.89 out of 4 [47]. A small number of parents (1.6% to 13.3%) cited the fact that HPV is an STI as a factor against HPV vaccination in two quantitative studies [35, 41], with parents from one qualitative study conditionally supporting vaccination for their daughter only if the child was not informed that the vaccine is against an STI [49]. There were no clear trends in disease perspectives reported by parents or young people over time.

#### Attitudes

Six quantitative studies assessed general vaccine attitudes [21, 35, 38, 39, 41, 45]. Three studies asked parents about the topics mentioned above (vaccine confidence, vaccine concerns, and disease perspective) and a mean score was created to evaluate the association of general attitudes with HPV vaccine acceptance [21, 35, 41]. Two of these studies showed parents with a positive attitude toward HPV vaccine were significantly more likely to accept HPV vaccination [21, 35]. However, the third study found no statistically significant association between attitude level and HPV vaccine acceptance [41]. Three studies focused on young people [38, 39, 45]. The first indicated that complete vaccination status was significantly associated with a “good attitude,” although this was not defined [38]. The second study showed positive attitude was predicted by younger age [45]. The last study compared attitudes between medical and nursing students, showing medical students had a better attitude (undefined) toward HPV vaccination [39]. The studies measuring attitudes among young people were too heterogeneous to indicate patterns across the years.

### Social process domain

#### Influence of religion

Seven studies assessed the influence of religion among parents [21, 35, 36, 41, 48, 49] and young people [50]. In two studies, parents cited religion as an influence of HPV vaccination decision-making process (range 11.3 to 44.1%) [35, 41]. The influence of religion increased over time, with a higher percentage of people citing it as important in the more recent study years. One study asked parents who rejected vaccination to identify their reasons why, and 12% cited the fact that HPV vaccination is not aligned with their religion beliefs [36]. Three quantitative studies [21, 35, 41] assessed association of parents’ religion with HPV vaccination acceptance, and none found a significant association. In two of the qualitative studies [48, 50], parents and young people said it was important to them that the vaccine was halal, with one parent saying: “The halal [permissible] factor is indeed influential… It has been through a long process and the safety is guaranteed by the government” [48]. However, one qualitative study reported that no parents had any concern that vaccination was haram (forbidden) according to Islamic law [49]. The growing concern of halal haram of HPV vaccine showed in the more recently published studies.

#### People who could influence vaccine decisions

Eight studies [21, 35, 36, 40, 41, 48–50] assessed people who could influence HPV vaccine decision-making. A range of people mentioned were spouse, other family members, parents, healthcare professionals, health cadres, schoolteachers, the children themselves, peers, the governor/the government, and no one. In three quantitative studies, healthcare providers were not the most influential people on HPV vaccine decision among parents, but spouses [35, 41] and teachers [36] were. Furthermore, one quantitative study of young people indicated no significant association between healthcare provider recommendation and adolescent girls’ self-efficacy to get HPV vaccination [40]. However, in one qualitative study healthcare providers remained reliable among parents since they had sufficient medical training [48].

One study of young people showed high parental support for HPV vaccination, with 91% parents allowing their daughter to get the vaccine [40]. The same study found that parental support was significantly associated with adolescent girls’ self-efficacy to obtain HPV vaccination [40]. This finding was also echoed by one qualitative study that found student’s willingness to obtain HPV vaccination depends on their parents [50]. One quantitative study [21] assessed subjective norms, defined as parents’ belief whether other people would approve or disapprove of HPV vaccination. The study found that positive subjective norms have strong association with parents’ decision to vaccinate their child against HPV [21].

Spouse was cited as the most influential person in two studies of parents (range 28.1 to 70.4%) [35, 41]; in one qualitative study, women showed conditional support for their children to have HPV vaccine if only they gained approval from their husband [49]. One qualitative study of parents showed that peers and the governor/government were also influential, with one parent saying: “I think the vaccine is good, because one of my friends also allowed her daughter to receive it.” [48].

### Motivation domain

Seven studies assessed motivation to get HPV vaccination, defined as acceptance, willingness, or intention to receive the vaccine themselves [42], or to vaccinate their children [21, 35, 36, 41, 48, 49]. Two studies reported high acceptance among parents (96.1%) and students (92%) [35, 42], and three additional studies reporting more than 70% of parents (range 70 to 91.3%) intended to get the vaccine for their children [21, 36, 48]. One qualitative study showed that parents would support HPV vaccination for their children [49]. Only one study reported low vaccine intention, with only 20% of parents in one area in West Java willing to vaccinate [41]. HPV vaccine acceptance, intention, and willingness among parents was lower in the more recent years compared to the earlier years of included studies.

### Practical issues domain

#### Affordability

Affordability of HPV vaccine was assessed in ten studies focused on parents [21, 35, 36, 41, 46, 48, 49, 51] and both on parents and young people [42, 50]. The cost parents face to take their daughter to take the HPV vaccine was identified as a barrier by parents and young people [21, 35, 36, 41, 42, 46, 50]. However, one quantitative study showed no students cited cost of vaccine as a barrier to take HPV vaccine [42]. Cost was identified as a barrier among parents throughout the years, though fewer parents identified this issue in more recent years. In one study, less than 15% parents could afford HPV vaccine [35], while in the other study, affordability was only cited as a reason to vaccinate by 11% of parents [41]. One qualitative study also reported this with one parent saying: “I don’t think I can afford it. I would rather to use the money to pay rent or to buy food” [48]. Despite the cost, four studies found parents were willing to pay for some or all of the vaccine [35, 41, 46, 49]. Up to 63% of parents were willing to pay partly of the HPV vaccination cost [35], but most parents still preferred for the vaccines to be free [50, 51]. The willingness to pay for HPV vaccination was higher in the earlier years, with a shifting preference for the vaccine to be free in more recent years. Two studies asked parents who they thought should be responsible for the cost of the HPV vaccine; more than 65% cited the government [35, 41].

#### Accessibility

Four studies of parents assessed accessibility as a factor for HPV vaccination [35, 36, 41, 48]. Two studies asked participants their preferred location for HPV vaccination, which was puskesmas (public healthcare centers) (range 20.6% to 64.9%) rather than schools [35, 41]. More than 90% parents were willing to get their daughter vaccinated against HPV at school in one quantitative study [36]. This was also reflected in one qualitative study: “I heard that they will get it in Year 5, so I was waiting for her to get it at school” [48]. Puskesmas was the preferred location for HPV vaccination in both earlier and later years, with a higher percentage in the earlier years. However, in the more recent years, there was an increase in the willingness to receive HPV vaccine at school.

## Discussion

This scoping review identified behavioral and social drivers of HPV vaccination among parents and young people in Indonesia through 18 studies with a combination of designs, and of mostly low quality. Many were quantitative studies and reported descriptive statistics without examining the association between HPV vaccine drivers and uptake. The most commonly reported drivers were related to what people think and feel about vaccines, while others including social process, motivation, and access barriers were less commonly reported.

Our review indicates low awareness and knowledge about HPV vaccination among parents and young people, with no clear trends over the years. A large systematic review including countries in Southeast Asia and West Pacific Region in 2018 also found low awareness and knowledge of cervical cancer, HPV infection and HPV vaccination [52]. Good awareness and knowledge are important for parents and young people to make an informed decision about HPV vaccination. Furthermore, having good awareness and knowledge may counteract misinformation regarding HPV vaccination [53]. Given the findings in our review, new strategies need to be employed to improve awareness and knowledge. Education strategies should deliver clear messages about the relationship between HPV infection and cervical cancer and the importance of HPV vaccine for prevention before sexual debut [54]. School-based vaccination programs may serve as an education platform to reach young people and parents [55, 56]. Young people are not fully autonomous decision-makers; they tend to follow their parents and be respectful of their parents’ authority [18, 57]. Therefore, it is important to ensure both parents and young people are well-informed about HPV vaccination and involved in shared decision-making for HPV vaccination.

Our review showed that while studies assessing the influence of religion did not frequently cite it as a factor in vaccine decision-making, there was an increase in its mention more recently. This finding is significant since religion has an important role in decision-making for other vaccines in Indonesia. A study exploring parents’ reason for incomplete childhood immunization revealed that parents of unimmunized children refuse vaccines because of their religious beliefs and parents tend to follow the religious leaders, which do not always support vaccines [58]. The halal-haram status of HPV vaccine was mentioned in our review with a growing concern in the more recent years of studies. Since the majority of the Indonesian population identifies as Muslim [59], concerns about vaccines that contain non-permissible animal-derived ingredients is growing [60]. Halal-haram considerations are cited to be an important factor in deciding to receive other vaccines, with haram status of the vaccine acting as barrier to uptake for rotavirus vaccine in Yogyakarta [61]. As the religious influence on HPV vaccine increased over time, the halal status of vaccines needs to be effectively communicated and involve religious authorities. The Indonesian Government successfully addressed this concern for the COVID-19 vaccine by involving the highest Muslim clerical council, the Indonesian Ulama Council (MUI) for COVID-19 vaccine certification, procurement, and quality assurance of vaccine production process [62]. Therefore, the involvement of religious authorities is needed to promote HPV vaccine.

Healthcare providers were not the most cited influencers in vaccine decision-making in our review, but spouses and teachers. This finding is in contrast with routine childhood vaccines in Indonesia [63, 64] and HPV vaccines in other countries [52, 65]. Healthcare providers are very influential in routine childhood vaccination in Indonesia, as children whose births assisted by healthcare workers are more likely to receive complete vaccination because they give vaccine recommendation to parents [63, 64]. A systematic review conducted in the United States showing that healthcare provider recommendation is associated with a higher HPV vaccine uptake [65]. Another review conducted in South East Asia and West Pacific Region that includes parents and young people also highlighted the important role of healthcare providers [52]. However, besides healthcare providers, other influential people include parents, family, peers, and teachers [52, 66]. These people were highly cited as influencers in our review. The role of fathers and extended family were influential for vaccine decisions because gender roles are an important issue in Indonesia where the society remains patriarchal [67, 68]. Mothers are supposed to care for children but do not necessarily have an autonomy to make vaccine decisions for their children [68, 69]. Therefore, in addition to healthcare providers, it is important to target influential people such as teachers, heads of households, and religious leaders with vaccine communication strategies. Empowerment of community influencers to become vaccine advocates can be impactful [70], especially teachers for the HPV vaccine [71]. A systematic review of behavioral vaccine interventions showed that training health and non-health vaccine champions can improve vaccine uptake [72].

The HPV vaccine is delivered primarily through schools. However, puskesmas was the preferred location in our review, probably because of the familiarity of puskesmas where parents got routine vaccination for their children. Nevertheless, our review showed a high willingness to get HPV vaccine in schools in the more recent years of studies when the HPV vaccination pilot programs had begun. School-based and facility-based HPV vaccination delivery is implemented in more than 50% of the HPV vaccination programs globally, where school-based implementation has shown a higher HPV vaccine uptake [73, 74]. Indonesia has implemented school-based immunization for other immunization other than HPV vaccine through Bulan Imunisasi Anak Sekolah, including measles-rubella (MR), diphtheria, and tetanus vaccines (DT and Td) [2, 75]. Despite the school-based program being more convenient and able to reach a wider population, there are challenges collecting parental consent. A systematic review that included high-income countries (HICs) conducted in 2014 shows that this is an important barrier in Australia, United Kingdom (UK), and the US [20]. Therefore, parental consent should be monitored as a potential barrier as the rollout of HPV vaccination progresses.

Concern about HPV vaccine cost was frequently mentioned in our review, with a decreasing trend in the later years of studies. This trend is likely due to the fact that initially, the vaccine was only offered for free to 5th and 6th grade students in schools within the piloted districts. For any others who wished to receive the HPV vaccine, the cost was around 1 million Indonesian Rupiah (60 USD) for one injection, which was considered expensive for most of the Indonesian population in low socioeconomic status [45, 49]. Cost of HPV vaccination has been shown as one of the most major barriers of HPV vaccination in countries where the vaccine is not funded by the government or public health insurance, including low-middle-income countries (LMICs) and HICs [24, 52, 76, 77]. A higher uptake of HPV vaccination is shown in countries with public funding, including Australia and UK [20, 76]. As the recent national expansion of the program provides the vaccine for free to all 5th and 6th grade girls, cost should not be a major consideration moving forward. However, indirect costs associated with vaccination should be considered such as transport and time off work.

### Recommendations

Although significant progress to improve the HPV vaccination program has been accomplished in Indonesia, several aspects need to be improved. Clear communication of halal-haram status of the HPV vaccine is needed to gain public confidence and increase the acceptance. Furthermore, parents, family, teachers, and other trusted community members need to be included in vaccine messaging as they are very influential. Educational interventions are critical to address low awareness and knowledge both for parents and young people. Lastly, ensuring vaccines are easily accessible, including through puskesmas and schools is important. School-based programs should be upscaled to cover out-of-school girls and manage the system to address girls who missed the school-based vaccination schedule.

Our findings suggest that there is still a knowledge gap regarding drivers of HPV vaccination in Indonesia that needs to be explored. High quality primary research in these areas is needed, particularly qualitative studies and studies that assess changes in drivers over time, such as pre- and post-vaccine introduction or early and later in the rollout of the HPV vaccination program. Studies to compare the drivers of HPV vaccine in different provinces in Indonesia are important since each province has a diverse culture and varying level of urban and rural areas. Furthermore, studies that assess association between the drivers and vaccine uptake are needed. Lastly, more studies need to include younger peoples’ views, as they are the primary beneficiaries of the HPV vaccination program.

### Strengths and limitations

The strengths of this review include the adherence to the established protocol for scoping review and the application of the BeSD framework to report study findings. There are several limitations. This review did not search for studies published in Bahasa Indonesia in specific Indonesian databases. However, we did not exclude studies in Bahasa Indonesia if they were identified. We also did not search the gray literature that may have provided important data not provided by published literature. The methods and measurement of the included studies meant it was not possible to conduct a meta-analysis, provide overall estimates of prevalence, or provide significance of different drivers. We acknowledge that the studies are not representative of all Indonesia provinces with most of the included studies conducted in provinces within Java Island. This limited geographic range may not reflect drivers experienced by parents and young people in other Indonesia provinces. Moreover, the study populations are not necessarily representative with younger people who are the target population of the national HPV vaccination program not generally included. Finally, the included studies have different quality levels of detail meaning some findings were less reliable than others.

## Conclusion

This review highlights the drivers of HPV vaccination among parents and young people in Indonesia. These findings are critical to optimize HPV vaccine uptake among young girls in Indonesia as the national HPV vaccine rollout is expanded, which will inform future HPV vaccination programs to improve vaccine service delivery and acceptance.

## Supplementary Information

Below is the link to the electronic supplementary material.

Supplementary file1 (PDF 63 KB)

Supplementary file2 (PDF 181 KB)

Supplementary file3 (PDF 134 KB)

Supplementary file4 (PDF 101 KB)

Supplementary file5 (PDF 81 KB)

## Data Availability

All data supporting the results are available within this manuscript and its supplementary materials.
