# Supplementary material for: The behavioral and social drivers of HPV vaccination among parents and young people in Indonesia: a scoping review
Source: Cancer Causes Control. 2025 Jul 2;36(11):1275–89. doi: 10.1007/s10552-025-02027-x (PMC12578677; doi:10.1007/s10552-025-02027-x)
Supplement: Supplementary file 2 — Supplementary file2 (PDF 181 KB) [file 10552_2025_2027_MOESM2_ESM.pdf]

The drivers of Human Papillomavirus (HPV) vaccination among parents and young people in Indonesia: A systematic review

To enable PROSPERO to focus on COVID-19 submissions, this registration record has undergone basic automated checks for eligibility and is published exactly as submitted. PROSPERO has never provided peer review, and usual checking by the PROSPERO team does not endorse content. Therefore, automatically published records should be treated as any other PROSPERO registration. Further detail is provided here.

Citation

Aisya Athifa, Jessica Kaufman, Margie Danchin. The drivers of Human Papillomavirus (HPV) vaccination among parents and young people in Indonesia: A systematic review. PROSPERO 2024 CRD42024525525 Available from: https://www.crd.york.ac.uk/prospero/display_record.php?ID=CRD42024525525

Review question

What are the drivers of HPV vaccination among parents and young people in Indonesia?

Searches

MEDLINE (Ovid), EMBASE, PubMed, Ovid Global Health

Types of study to be included

We will include qualitative, quantitative, and mixed-method studies.

Condition or domain being studied

Behavioural and social drivers of HPV vaccination in Indonesia

Participants/population

Participants are parents and young people under 24 years old in Indonesia

Intervention(s), exposure(s)

HPV vaccination

Comparator(s)/control

Not applicable

Context

The review will include published studies conducted in Indonesia with the English language and Bahasa Indonesia. Studies conducted in one more country will be included if the data about or from Indonesia can be separated. There will be no limitation on the publication dates of the studies.

Main outcome(s)

Behavioural and social drivers including motivation, thoughts and feelings, social processes, and practical issues of HPV vaccination from the perspective of parents and young people.

Measures of effect

Numbers and percentages for frequency, odds ratio for association, and description of thematic data

Additional outcome(s)

None

Measures of effect

None

Data extraction (selection and coding)

A data extraction template will be developed. Extracted data including authors, year of publication, study setting, study design, participant characteristics, sample size, data on the behavioural and social drivers, study limitation, and risk of bias. Screening and selection process will be conducted by two people, to screen the title, abstract, and full text.

Risk of bias (quality) assessment

Two people will assess the risks of bias for each study using the Mixed Method Appraisal tool (MMAT). Any disagreement will be settled with discussion and the help of a third review author.

Strategy for data synthesis

We will use narrative synthesis to present the findings of this review. The data will be categorised based on study design, population, and results. The data will be presented in a descriptive format.

Analysis of subgroups or subsets

We plan to compare different study designs (quantitative, qualitative, and mixed methods) and participant characteristics (parents and young people under 24 years old) if there are sufficient studies and data.

Contact details for further information

Aisya Athifa

aisya.athifa@mcri.edu.au

Organisational affiliation of the review

The University of Melbourne

https://www.unimelb.edu.au/

Review team members and their organisational affiliations

Miss Aisya Athifa. University of Melbourne

Dr Jessica Kaufman. Vaccine Uptake Group, Murdoch Children's Research Institute

Professor Margie Danchin. Vaccine Uptake Group, Murdoch Children's Research Institute

Type and method of review

Narrative synthesis, Systematic review

Anticipated or actual start date

20 March 2024

Anticipated completion date

07 June 2024

Funding sources/sponsors

None

Conflicts of interest

Language

English

Country

Australia

Stage of review

Review Ongoing

Subject index terms status

Subject indexing assigned by CRD

Subject index terms

MeSH headings have not been applied to this record

Date of registration in PROSPERO

30 March 2024

Date of first submission

19 March 2024

Details of any existing review of the same topic by the same authors

None

Stage of review at time of this submission

The review has not started

Stage	Started	Completed
Preliminary searches	No	No
Piloting of the study selection process	No	No
Formal screening of search results against eligibility criteria	No	No
Data extraction	No	No
Risk of bias (quality) assessment	No	No
Data analysis	No	No

The record owner confirms that the information they have supplied for this submission is accurate and complete and they understand that deliberate provision of inaccurate information or omission of data may be construed as scientific misconduct.

The record owner confirms that they will update the status of the review when it is completed and will add publication details in due course.

Versions

30 March 2024

30 March 2024
