# Supplementary material for: The behavioral and social drivers of HPV vaccination among parents and young people in Indonesia: a scoping review
Source: Cancer Causes Control. 2025 Jul 2;36(11):1275–89. doi: 10.1007/s10552-025-02027-x (PMC12578677; doi:10.1007/s10552-025-02027-x)
Supplement: Supplementary file 3 — Supplementary file3 (PDF 134 KB) [file 10552_2025_2027_MOESM3_ESM.pdf]

Supplementary File 3 - The search strategies, number of articles found, and search date for each database

**Database**	**Search Terms (English)**
MEDLINE<br><br>Date of Search: 13 March 2024<br><br>Number of Articles: 49	1. exp Papillomavirus Infections/<br>2. papillomaviridae/ or exp alphapapillomavirus/ or exp human papillomavirus viruses/<br>3. (papillomaviridae or txid151340 or alphapapillomavirus* or alpha-papillomavirus* or human-papillomavirus* or human-papilloma-virus or HPV or HPV16* or papilloma-viral or papillomaviral).tw,kf.<br>4. Uterine neoplasms/ or uterine cervical neoplasms/<br>5. (uterine-cancer* or uterine-neoplasm* or cervical-cancer* or cervical-neoplasm* or cancer-of-the-cervix* or cancer-of-the-uterine* or neoplasm-of-the-cervix* or neoplasm-of-the-uterine* or uterine-tumor* or uterine-tumorigenes* or uterine-tumour* or uterus-neoplasia* or uterus-neoplasm* or uterus-tumour* or uterus-tumor* or neoplasia-of-the-uterus or neoplasm-of-the-uterus or neoplastic-uterine or tumor-of-the-uterus or tumour-of-the-uterus or cervical-neoplas* or cervical-tumor* or cervical-tumorigenes* or cervical-tumour* or cervix-neoplas* or cervix-tumor* or cervix-tumorigenes* or cervix-tumour* or uteri-tumor* or uteri-tumour* or neoplasia-of-the-cervix or neoplasm-of-the-cervix or neoplastic-cervical or neoplastic-cervix or tumor-of-the-cervix or tumour-of-the-cervix or tumor-of-the-uterine or tumour-of-the-uterine).tw,kf.<br>6. 1 or 2 or 3 or 4 or 5<br>7. Indonesia/<br>8. (Aceh or Bali or West-Nusa-Tenggara or East-Nusa-Tenggara or Kalimantan or Sulawesi or Gorontalo or Maluku or Papua or Celebes or dutch-new-guinea or east-indies or Indonesia* or Irian-barat or Jambi or Bangka-Belitung-Island* or Bengkulu or Lampung or Banten or Jakarta or Java or Madoera or Madura or malay-archipelago or Netherlands-new-guinea or Riau or Sumatra or Sumatera or Timor or western-new-guinea or west-irian or west-new-guinea or Yogyakata).tw,kf.<br>9. 7 or 8<br>10. exp immunization/<br>11. exp Immunization Programs/<br>12. (vaccin* or immunis* or immuniza*).tw,kf.<br>13. 10 or 11 or 12<br>14. 6 and 9 and 13
EMBASE<br><br>Date of Search: 20 March 2024<br><br>Number of Articles: 76	1. exp papillomavirus infection/<br>2. exp papillomaviridae<br>3. Wart virus/<br>4. (papillomaviridae or txid151340 or alphapapillomavirus* or human-papillomavirus* or human-papilloma-virus* or HPV or HPV16* or papilloma-viral or papillomaviral).tw,kf,dq.<br>5. (uterine-cancer* or uterine-neoplasm* or cervical-cancer* or cervical-neoplasm* or cancer-of-the-cervix* or cancer-of-the-uterine* or neoplasm-of-cervix* or neoplasm-of-the-uterine* or uterine-tumor* or uterine-tumorigenes* or uterine-tumour* or uterus-neoplasia* or uterus-neoplasm* or uterus-tumour* or uterus-

Database	Search Terms (English)
	tumor* or neoplasia-of-the-uterus or neoplasm-of-the-uterus or neoplastic-uterine or tumor-of-the-uterus or tumour-of-the-uterus).tw,kf.<br>6. Exp uterus tumor<br>7. (uterine-cancer* or uterine-neoplasm* or cervical-cancer* or cervical-neoplasm* or cancer-of-the-cervix* or cancer-of-the-uterine* or neoplasm-of-the-cervix* or neoplasm-of-the-uterine* or uterine-tumor* or uterine-tumorigenes* or uterine-tumour* or uterus-neoplasia* or uterus-neoplasm* or uterus-tumour* or uterus-tumor* or neoplasia-of-the-uterus or neoplasm-of-the-uterus or neoplastic-uterine or tumor-of-the-uterus or tumour-of-the-uterus or cervical-neoplas* or cervical-tumor* or cervical-tumorigenes* or cervical-tumour* or cervix-neoplas* or cervix-tumor* or cervix-tumorigenes* or cervix-tumour* or uteri-tumor* or uteri-tumour* or neoplasia-of-the-cervix or neoplasm-of-the-cervix or neoplastic-cervical or neoplastic-cervix or tumor-of-the-cervix or tumour-of-the-cervix or tumor-of-the-uterine or tumour-of-the-uterine).tw,kf,dq.<br>8. Exp uterine cervix tumor/<br>9. 1 or 2 or 3 or 4 or 5 or 6 or 7 or 8<br>10. Exp Indonesia/<br>11. (Aceh or Bali or West-Nusa-Tenggara or East-Nusa-Tenggara or Kalimantan or Sulawesi or Gorontalo or Maluku or Papua or Celebes or dutch-new-guinea or east-indies or Indonesia* or Irian-barat or Jambi or Bangka-Belitung-Island* or Bengkulu or Lampung or Banten or Jakarta or Java or Madoera or Madura or malay-archipelago or Netherlands-east-indies or Netherlands-new-guinea or Riau or Sumatra or Sumatera or Timor or western-new-guinea or west-irian or west-new-guinea or Yogyakarta).tw,kf,dq.<br>12. 10 or 11<br>13. exp immunization/<br>14. exp preventice health service/<br>15. (vaccin* or immunis* or immuniz*).tw,kf,dq.<br>16. 13 or 14 or 15<br>17. 9 and 12 and 16
PubMed<br><br>Date of search: 20 March 2024<br><br>Number of Articles: 28	#1 title/abstract<br>“papillomaviridae” OR “txid151340” OR “alphapapillomavirus*” OR “alpha-papillomavirus*” OR “papillomavirus*” OR “papilloma-virus*” OR “HPV” OR “HPV16*” OR “papilloma-viral” OR “papillomaviral”<br>#2 title/abstract<br>“uterine-cancer*” OR “uterine-neoplasm*” OR “cervical-cancer*” OR “cervical-neoplasm*” OR “cancer-of-the-cervix*” OR “cancer-of-the-uterine*” OR “neoplasm-of-the-cervix*” OR “neoplasm-of-the-uterine*” OR “uterine-tumor*” OR “uterine-tumorigenes*” OR “uterine-tumour*” OR “uterus-neoplasia*” OR “uterus-neoplasm*” OR “uterus-tumour*” OR “uterus-tumor*” OR “neoplasia-of-the-uterus” OR “neoplasm-of-the-uterus” OR “neoplastic-uterine” OR “tumor-of-the-uterus” OR “tumour-of-the-uterus” OR “cervical-neoplas*” OR “cervical-tumor*” OR “cervical-tumorigenes*” OR “cervical-tumour*” OR “cervix-neoplas*” OR “cervix-tumor*” OR “cervix-tumorigenes*” OR “cervix-tumour*” OR “uteri-tumor*” OR “uteri-tumour*” OR “neoplasia-of-the-cervix” OR “neoplasm-of-the-cervix” OR “neoplastic-cervical” OR “neoplastic-cervix” OR “tumor-of-the-cervix” OR “tumour-of-the-cervix” OR “tumor-of-the-uterine” OR “tumour-of-the-uterine” OR “uterine-cervical-neoplasm*” OR “uterine-cervical-cancer*”<br>#3 #1 OR #2

Database	Search Terms (English)
	#4 title/abstract<br>“Aceh” OR “Bali” OR “West-Nusa-Tenggara” OR “East-Nusa-Tenggara” OR “Kalimantan” OR “Sulawesi” OR “Gorontalo” OR “Maluku” OR “Papua” OR “Celebes” OR “dutch-new-guinea” OR “east-indies” OR “Indonesia*” OR “Irian-barat” OR “Jambi” OR “Bangka-Belitung-Island*” OR “Bengkulu” OR “Lampung” OR “Banten” OR “Jakarta” OR “Java” OR “Madoera” OR “Madura” OR “malay-archipelago” OR “Netherlands-east-indies” OR “Netherlands-new-guinea” OR “Riau” OR “Sumatra” OR “Sumatera” OR “Timor” OR “western-new-guinea” OR “west-irian” OR “west-new-guinea” OR “Yogyakarta”<br>#5 title/abstract<br>“vaccin*” OR “immunis*” OR “immuniz*”<br>#6 **all fields**<br>NOTNLM OR publisher[sb] OR inprocess[sb] OR pubmednotmedline[sb] OR indatareview[sb] OR pubstatusaheadofprint<br>#7 #3 AND #4 AND #5 AND #6
Ovid Global Health<br><br>Date of search: 20 March 2024<br><br>Number of Articles: 38	1. exp papillomaviridae/<br>2. (papillomaviridae or txid151340 or alphapapillomavirus* or human-papillomavirus* or human-papilloma-virus* or HPV or HPV16* or papilloma-viral or papillomaviral).ti,ab.<br>3. exp uterine cancer/<br>4. exp cervical cancer/<br>5. (uterine-cancer* or uterine-neoplasm* or cervical-cancer* or cervical-neoplasm* or cancer-of-the-cervix* or cancer-of-the-uterine* or neoplasm-of-the-cervix* or neoplasm-of-the-uterine* or uterine-tumor* or uterine-tumorigenes* or uterine-tumour* or uterus-neoplasia* or uterus-neoplasm* or uterus-tumour* or uterus-tumor* or neoplasia-of-the-uterus or neoplasm-of-the-uterus or neoplastic-uterine or tumor-of-the-uterus or tumour-of-the-uterus or cervical-neoplas* or cervical-tumor* or cervical-tumorigenes* or cervical-tumour* or cervix-neoplas* or cervix-tumor* or cervix-tumorigenes* or cervix-tumour* or uteri-tumor* or uteri-tumour* or neoplasia-of-the-cervix or neoplasm-of-the-cervix or neoplastic-cervical or neoplastic-cervix or tumor-of-the-cervix or tumour-of-the-cervix or tumor-of-the-uterine or tumour-of-the-uterine).ti,ab.<br>6. 1 or 2 or 3 or 4 or 5<br>7. exp Indonesia/<br>8. (Aceh or Bali or West-Nusa-Tenggara or East-Nusa-Tenggara or Kalimantan or Sulawesi or Gorontalo or Maluku or Papua or Celebes or dutch-new-guinea or east-indies or Indonesia* or Irian-barat or Jambi or Bangka-Belitung-Island* or Bengkulu or Lampung or Banten or Jakarta or Java or Madoera or Madura or malay-archipelago or Netherlands-east-indies or Netherlands-new-guinea or Riau or Sumatra or Sumatera or Timor or western-new-guinea or west-irian or west-new-guinea or Yogyakarta).ti,ab.<br>9. 7 or 8<br>10. Exp immunization/<br>11. Immunization programmes/<br>12. (vaccin* or immunis* or immuniz*).ti,ab<br>13. 10 or 11 or 12<br>14. 6 and 9 and 13
