# Supplementary material for: The behavioral and social drivers of HPV vaccination among parents and young people in Indonesia: a scoping review
Source: Cancer Causes Control. 2025 Jul 2;36(11):1275–89. doi: 10.1007/s10552-025-02027-x (PMC12578677; doi:10.1007/s10552-025-02027-x)
Supplement: Supplementary file 4 — Supplementary file4 (PDF 101 KB) [file 10552_2025_2027_MOESM4_ESM.pdf]

Supplementary File 4 - Data Extraction Template

No	Review Title	Study ID	Language	Country/ies	Study Location		Year of study	Time period	HPV vaccine/routine vaccine	Publication type	Study design	Study aim	Study participants	Sample size
					Provinces	Urban/rural/both								

Data extraction template continued.

Measure of effect	Outcomes categorised based on WHO BeSD domain										Others (outside WHO BeSD domain)	HPV vaccine uptake	Limitation of studies	Notes
	Thinking and Feeling					Social Process		Motivation or hesitancy	Practical Issues					
	Awareness	Knowledge	Attitudes	Health beliefs	Vaccine confidence	People who could influence vaccine decision	Influence of religion		Affordability	Accessibility				
Frequency														
Measurement scales used														
Association														
Description of themes and relevant quotes														
