# Supplementary material for: The behavioral and social drivers of HPV vaccination among parents and young people in Indonesia: a scoping review
Source: Cancer Causes Control. 2025 Jul 2;36(11):1275–89. doi: 10.1007/s10552-025-02027-x (PMC12578677; doi:10.1007/s10552-025-02027-x)
Supplement: Supplementary file 5 — Supplementary file5 (PDF 81 KB) [file 10552_2025_2027_MOESM5_ESM.pdf]

Supplementary File 5 - Excluded Studies and Reasons

No	Studies – Author (year published)	Reasons
1	Putri (2022)	Wrong patient population
2	Febriani (2018)	Wrong patient population
3	Winarto (2022)	Wrong patient population
4	Fitriani (2018)	Wrong patient population
5	Khatiwada (2021)	Wrong patient population
